# Maternal Urinary Metal and Metalloid Concentrations in Association with Oxidative Stress Biomarkers

**DOI:** 10.3390/antiox10010114

**Published:** 2021-01-15

**Authors:** Pahriya Ashrap, Deborah J. Watkins, Ginger L. Milne, Kelly K. Ferguson, Rita Loch-Caruso, Jennifer Fernandez, Zaira Rosario, Carmen M. Vélez-Vega, Akram Alshawabkeh, José F. Cordero, John D. Meeker

**Affiliations:** 1 Department of Environmental Health Sciences, University of Michigan School of Public Health, Ann Arbor, MI 48109, USA; pahriya@umich.edu (P.A.); debjwat@umich.edu (D.J.W.); rlc@umich.edu (R.L.-C.); jafernan@umich.edu (J.F.); 2 Vanderbilt University Medical Center, Division of Clinical Pharmacology, Nashville, TN 37232, USA; ginger.milne@vumc.org; 3 Epidemiology Branch, National Institute of Environmental Health Sciences, Research Triangle Park, North Carolina, NC 27709, USA; kelly.ferguson2@nih.gov; 4 Department of Epidemiology and Biostatistics, University of Georgia, Athens, GA 30602, USA; zaira.rosario@upr.edu (Z.R.); jcordero@uga.edu (J.F.C.); 5 UPR Medical Sciences Campus, University of Puerto Rico Graduate School of Public Health, San Juan, PR 00921, USA; carmen.velez@upr.edu; 6 College of Engineering, Northeastern University, Boston, MA 02115, USA; a.alshawabkeh@northeastern.edu

**Keywords:** metals, isoprostane, biomarkers, oxidative stress, Puerto Rico

## Abstract

Metal exposure has been associated with a wide range of adverse birth outcomes and oxidative stress is a leading hypothesis of the mechanism of action of metal toxicity. We assessed the relationship between maternal exposure to essential and non-essential metals and metalloids in pregnancy and oxidative stress markers, and sought to identify windows of vulnerability and effect modification by fetal sex. In our analysis of 215 women from the PROTECT birth cohort study, we measured 14 essential and non-essential metals in urine samples at three time points during pregnancy. The oxidative stress marker 8-iso-prostaglandin F2α (8-iso-PGF2α) and its metabolite 2,3-dinor-5,6-dihydro-15-15-F2t-IsoP, as well as prostaglandin F2α (PGF2α), were also measured in the same urine samples. Using linear mixed models, we examined the main effects of metals on markers of oxidative stress as well as the visit-specific and fetal sex-specific effects. After adjustment for covariates, we found that a few urinary metal concentrations, most notably cesium (Cs) and copper (Cu), were associated with higher 8-iso-PGF2α with effect estimates ranging from 7.3 to 14.9% for each interquartile range, increase in the metal concentration. The effect estimates were generally in the same direction at the three visits and a few were significant only among women carrying a male fetus. Our data show that higher urinary metal concentrations were associated with elevated biomarkers of oxidative stress. Our results also indicate a potential vulnerability of women carrying a male fetus.

## 1. Introduction

Metals are environmental contaminants with the potential to impact biological pathways that contribute to preterm delivery [1,2,3,4,5]. One of the leading proposed mechanisms for metal toxicity is oxidative stress, defined as the homeostatic imbalance between cellular oxidants and availability of antioxidants to favor oxidation [6]. Oxidative stress plays an important role in the development of many adverse birth outcomes, including preeclampsia, preterm birth, and intrauterine growth restriction [7,8,9,10,11]. The levels of oxidative stress biomarkers, such as 8-iso-prostaglandin F2α (8-iso-PGF2α), increase during pregnancy and peak at delivery [12], suggesting that this mechanism plays an important role in normal childbirth. Previous human studies have shown positive associations between higher levels of oxidative stress biomarkers (8-iso-PGF2α) and preterm birth [13,14,15,16,17]. A recent analysis in the Puerto Rico Testsite for Exploring Contamination Threats (PROTECT) cohort study also suggested that elevated levels of 8-iso-PGF2α and its metabolite are associated with higher odds of overall preterm birth and particularly spontaneous preterm birth [18].

Several in vivo and in vitro studies have linked metal exposure with increased formation of reactive oxygen species (ROS) [19,20]. The excessive ROS can induce oxidative stress and cause damage to cells, leading to the release of lipid peroxidation products into circulation [21]. Elevated biomarkers of oxidative stress in association with exposure to heavy metals, including lead (Pb), arsenic (As), and cadmium (Cd), have been reported [22,23,24,25,26]. These non-essential metals have no known physiologic role in the human body and can be toxic if present even at low concentrations [1,27,28]. Moreover, they have been associated with preterm birth in epidemiological studies [29,30,31,32,33,34,35,36,37], including studies in the PROTECT cohort, where Pb, even at low-levels, was the most strongly associated with risk of preterm birth [38]. However, essential metals, such as copper (Cu), iron (Fe), manganese (Mn), molybdenum (Mo), selenium (Se), and zinc (Zn), which are important for human health, as well as other metals, such as cesium (Cs) and antimony (Sb), that are not classified as essential or non-essential, remain understudied [39,40]. Most metals, including essential metals, are redox-active and therefore have the potential to increase production of ROS and enhance lipid peroxidation [41,42]. To our knowledge, two prior studies explored the direct effects of essential and non-essential trace metals on oxidative stress biomarkers during pregnancy [26,43]. Dashner-Titus et al. reported that As is associated with increased levels of urinary 8-iso-PGF2α [26] and Kim et al. found positive associations of urinary Se and Cu with oxidative stress markers [43]. Whereas both prior studies were cross-sectional, the PROTECT study provided an opportunity to explore the longitudinal effect of various essential and non-essential metals on oxidative stress. The objective of this study was to explore the association between urinary metals and oxidative stress biomarkers, as well to identify windows of vulnerability and effect modification by fetal sex, by utilizing repeated measures of biomarkers among pregnant women participating in PROTECT.

## 2. Materials and Methods

### 2.1. Study Population

This study used data collected from women participating in the PROTECT study, an ongoing, prospective birth cohort [44,45,46,47]. The PROTECT study launched in 2010 with funding from the National Institute of Environmental Health Sciences (NIEHS) Superfund Research Program and is conducted in Puerto Rico because of its high preterm birth rate and the extent of hazardous waste contamination on the island. PROTECT aims to explore environmental exposures and other factors contributing to preterm birth risk and other adverse birth outcomes in Puerto Rico. 

**Figure 1:**
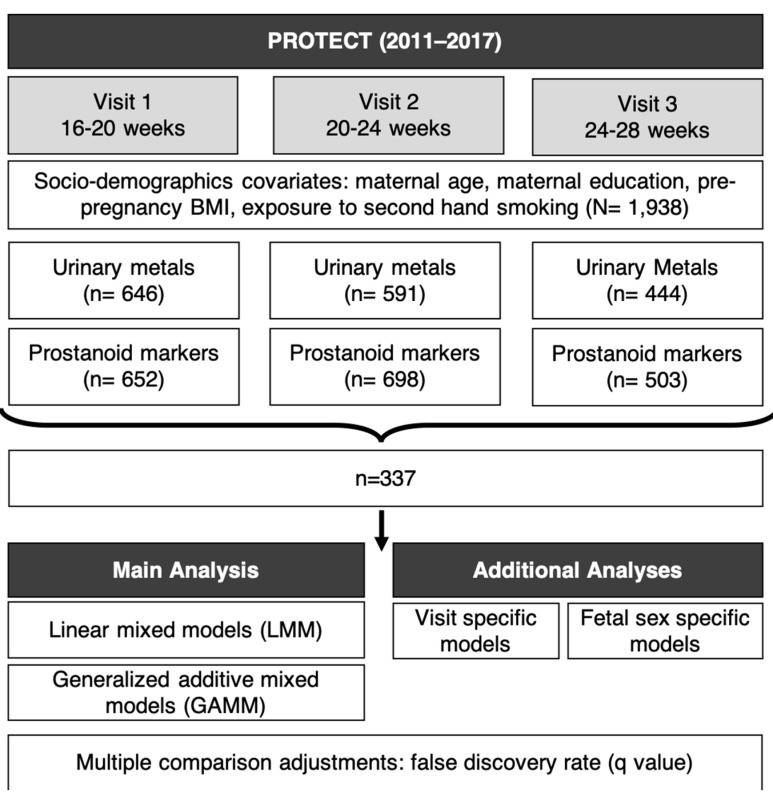
Schematic plot for PROTECT cohort study design, sample size, and statistical methods.

Study participants were recruited at approximately 14 ± 2 weeks of gestation at seven prenatal clinics and hospitals throughout Northern Puerto Rico and followed until delivery [44,45]. Inclusion criteria for this study were: maternal age between 18 and 40 years; residence inside of the Northern Karst aquifer region; disuse of oral contraceptives during the three months prior to pregnancy; disuse of in vitro fertilization to become pregnant; and free of any major medical or obstetrical complications, including pre-existing conditions, such as diabetes, hypertension, etc. Each woman participated in a total of up to three study visits (18 ± 2 weeks, 22 ± 2 weeks, and 26 ± 2 weeks of gestation). Detailed information on medical and pregnancy history were collected at the initial visit. During an in-home visit (second visit), nurse-administered questionnaires were used to gather information on housing characteristics, employment status, and family situation. Spot urine samples were collected from women at up to three visits. The present analysis reflects 337 urine samples from 215 women with measured metal(loid) and oxidative stress biomarker concentrations (Figure 1).

The research protocol was approved by the Ethics and Research Committees of the University of Puerto Rico and participating clinics, the University of Michigan, Northeastern University, and the University of Georgia (Approval number: A8570110). The study was described in detail to all participants, and informed consent was obtained prior to study enrollment.

### 2.2. Measurement of Metals

Spot urine was collected in sterile polypropylene cups and aliquoted within one hour after collection. All samples were frozen and stored at −80 °C and shipped on dry ice. Analysis was performed at NSF International (Ann Arbor, MI, USA), where concentrations of 21 metals and metalloids were measured: Arsenic (As), barium (Ba), beryllium (Be), cadmium (Cd), cobalt (Co), chromium (Cr), cesium (Cs), copper (Cu), mercury (Hg), manganese (Mn), molybdenum (Mo), nickel (Ni), lead (Pb), platinum (Pt), Sb, tin (Sn), titanium (Ti), tungsten (W), uranium (U), vanadium (V), and zinc (Zn). Metal(loid) concentrations were measured using inductively-coupled plasma mass spectrometry (ICPMS) as described previously [48]. Considering that biological samples have high levels of carbon and chloride in the matrix, the laboratory selected the appropriate isotopes for the requested elements to best avoid interferences where possible. The ICPMS was calibrated with a blank and a minimum of four standards for each element of interest. An R^2^ value of >0.995 was the minimum criteria for an acceptable calibration curve. The calibration curves were verified by initial checks at three calibration points within the curve. Continuing calibration checks and blanks after every 10 samples were also utilized throughout the analytical run to ensure the ICPMS system was maintaining acceptable performance. Urinary specific gravity was measured at the University of Puerto Rico Medical Sciences Campus using a hand-held digital refractometer (Atago Co., Ltd., Tokyo, Japan) as an indicator of urine dilution.

### 2.3. Measurement of Oxidative Stress Biomarkers

Urine samples were collected in polypropylene containers, divided into aliquots, and frozen at −80 °C until analysis [47]. To assess oxidative stress, the following prostanoids were measured in urine samples: 8-iso-PGF2α, the 8-iso-PGF2α major metabolite 2,3-dinor-5,6-dihydro-15-15-F2t-IsoP, and PGF2α. Analyses were performed by the Eicosanoid Core Laboratories at the Vanderbilt University Medical Center (Nashville, TN, USA). All three prostanoids were quantified using the GC/NICI-MS on an Agilent 5973 inert mass selective detector that is coupled with an Agilent 6890n Network GC system (Agilent Labs, Torrance, CA, USA) [49,50,51]. The precision of this assay in biological fluids is +6% and the accuracy is 94%. Further details describing the measurement of oxidative stress biomarker concentrations are available elsewhere [51].

Although 8-iso-PGF2α has been used as a biomarker of oxidative stress and its release attributed to chemical (nonenzymatic) lipid peroxidation [52,53], it may not solely be a biomarker of oxidative stress because 8-iso-PGF2α is also produced by prostaglandin-endoperoxide synthases (PGHS)-mediated enzymatic lipid peroxidation [54,55]. Enzymatic lipid peroxidation is significantly induced in inflammation, which can occur as a consequence or stimulator of oxidative stress [56,57]. Thus, the fractions of 8-iso-PGF2α contributed from chemical lipid peroxidation and enzymatic lipid peroxidation were used to distinguish and quantify the contribution of the two pathways [18,26,58]. This method was previously introduced and described in detail by van’t Erve et al. [54] and has been supported in an animal model to distinguish biomarker synthesis pathways [55]. Therefore, in this analysis, we additionally examined the hypothesized chemical fraction of 8-iso-PGF2α, which reflects the amount of 8-iso-PGF2α attributable to chemical lipid peroxidation, as well as the hypothesized enzymatic fraction of 8-iso-PGF2α, the amount attributable to inflammation induced enzymatic lipid peroxidation. The fractions were calculated using the ratio of 8-iso-PGF2α to PGF2α as described in detail by van‘t Erve et al. [54].

### 2.4. Statistical Methods

Metal and oxidative stress biomarker concentrations below the limit of detection (LOD) were replaced by LOD/√2. For statistical analysis, we included metal(loid)s with at least 70% of samples having concentrations above the LOD as continuous variables. Samples with very low detection rates (<30%) of metals, including the metals Be, Cr, Ti, U, V, Pt, and W, were excluded from the analyses. Descriptive statistics were calculated for all exposure and outcome variables. Distributions of all urinary metals and oxidative stress biomarkers were right skewed and, thus, were natural log transformed for all analyses.

We used linear mixed models (LMM) with a random intercept for subject ID to model each prostanoid measure as the dependent variable, with separate models for each exposure biomarker. The crude models included the metal concentration as the exposure and specific gravity as a covariate to adjust for urinary dilution [48,59,60]. The final set of covariates were selected based on a priori knowledge and if their inclusion appreciably changed the effect estimates of metal exposure. The covariates considered were study visit, maternal age, insurance type, maternal education level (an indicator of socioeconomic status), marital status, employment status, gravidity, pre-pregnancy body mass index (BMI), smoking, exposure to second-hand smoking, alcohol consumption, and gestational age at the time of sample collection. The final models were controlled for study visit, maternal age, maternal education level, marital status, pre-pregnancy BMI, and exposure to second-hand smoking.

We conducted additional analyses to assess potential windows of vulnerability in pregnancy. We included interaction terms between metal concentrations and each visit indicator separately into the LMMs to obtain visit-specific metal effect estimates. In these separate models the effect estimates of the covariates were still assessed using the whole dataset with the LMM structures rather than a subset of the dataset as in a stratified analysis. Furthermore, we considered the possibility of differential vulnerability among pregnant women carrying a male fetus vs. a female fetus. Therefore, to understand whether the effect estimates for metals on maternal oxidative stress differed according to infant sex, all single-pollutant models were refitted with the addition of an interaction term between metal concentrations and infant sex indicator, and the interaction term coefficient was tested for significance.

Finally, we used adjusted generalized additive mixed models (GAMM) to graphically depict the relationship between metal concentrations and oxidative stress markers. The results were presented as change in oxidative stress biomarkers (95% confidence intervals per interquartile range (IQR) increase in metal concentrations. We also considered significance after adjusting for multiple testing using the Benjamini–Hochberg method [61]. Since oxidative stress biomarkers were correlated, we calculated *q* values (adjusted *p* values) treating each outcome as a family of tests (14 tests per outcome). A cutoff of 0.05 for *q* value was used to further interpret main results with greater confidence. Data were analyzed using R version 3.6.2 [62].

## 3. Results

### 3.1. Demographics

Demographic characteristics of 215 women in this analysis are summarized in Table 1 and were described previously [46,63]. Briefly, the cohort included primarily non-smokers (81%) in their late 20s (median = 27 years) with half of the women having a BMI less than 25 kg/m^2^ prior to pregnancy. The majority of women (58%) had private medical insurance and were employed. More than half of them had annual household incomes less than $30,000 while 76% had reported graduating from college or higher. Very few (6%) of the women reported consumption of alcohol within the last few months.

### 3.2. Descriptive Statistics

Descriptive statistics (geometric mean, geometric standard deviation, select percentiles) of urinary metals and markers of oxidative stress are presented in Table 2. Urinary metals and oxidative stress biomarkers were measured among 215 women in up to three repeated urine samples (visit 1 = 124, visit 2 = 123, visit 3 = 91). Spearman correlations between different metals [64] and distribution of oxidative stress markers [18] were previously reported in detail. Briefly, levels of most urinary metals in pregnant Puerto Rican women were higher than levels observed in nonpregnant women ages 18–40 in the general U.S. population [64]. All of the women in our study had essential metal concentrations (Mn and Zn) within the normal physiological range [65,66] and none of the non-essential metal concentrations (Hg and Pb) exceed the level of concern [67,68]. A few moderate to strong correlations between urinary metals (Pb and Ba, R = 0.47; Cd and Pb, R = 0.55, Ni and Co, R = 0.55; Ni and Ba, R = 0.59) were observed. SG-corrected urinary concentrations of metal(loid)s were significantly different between the three visits for Co, Cs, Cu, Mo, and Zn (*p* < 0.05 for all). The geometric mean concentrations of 8-iso-PGF2α and the 8-iso-PGF2α metabolite were 1.8 ng/mL and 0.91 ng/mL, respectively, and were moderately correlated (Spearman R = 0.67, *p*-value < 0.01). PGF2α had a geometric mean concentration of 2.8 ng/mL and was also moderately associated with 8-iso-PGF2α (Spearman R = 0.74, *p*-value < 0.01) and the 8-iso-PGF2α metabolite (Spearman R = 0.56, *p*-value < 0.01).

### 3.3. Urinary Metals and Prostanoids

**Figure 2:**
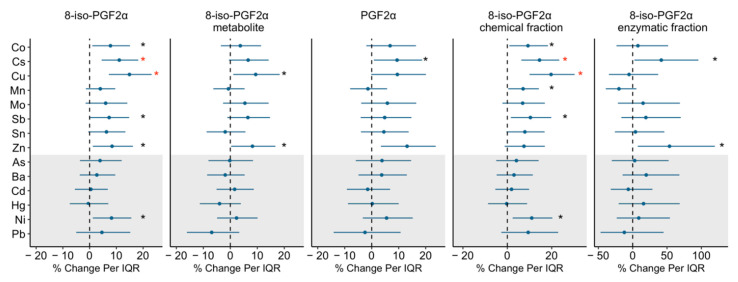
Percent change in prostanoids associated with urinary metal concentrations. Effect estimates presented as percent change (%) for interquartile range (IQR) increase in exposure biomarker concentration. Models were adjusted for study visit, maternal age, maternal education, marital status, pre-pregnancy BMI, and exposure to secondhand smoking. Abbreviations: cobalt (Co); cesium (Cs); copper (Cu); manganese (Mn); molybdenum (Mo); antimony (Sb); tin (Sn); zinc (Zn); arsenic (As); barium (Ba); cadmium (Cd); mercury (Hg); nickel (Ni); lead (Pb). White shading indicates essential metals and grey shading indicates non-essential metals. Since Cs is not regarded as essential to the health of plants or animals nor does it present a hazard to them, Cs was considered as essential metal for the analysis. Black * denotes *p* value < 0.05; red * denotes a *p* value < 0.05 and *q* value (false discovery rate) < 0.05.

The full models included 314 samples which had complete data on the adjusted covariates (study visit, maternal age, maternal education level, marital status, pre-pregnancy BMI, and exposure to second-hand smoking). Figure 2 presents the associations between urinary metal concentrations and prostanoid markers, and effect estimates, confidence intervals, and *p* values are also given in Supplemental Table S1.

As presented in Figure 2, the effect estimates from most models on urinary metals were positive. In adjusted models, several urinary metals, including, the essential metals Co, Cu, and Zn, the non-essential metal Ni, and Cs and Sb (not classified as essential or non-essential) were significantly associated with increased 8-iso-PGF2α, the effect estimates ranging from 7.3–14.9% increased 8-iso-PGF2α levels per IQR increase in the metal concentration. When we examined these associations for the enzymatic and chemical fractions of 8-iso-PGF2α, similar significant positive associations remained for the metals and the chemical fraction of 8-iso-PGF2α. The enzymatic fraction of 8-iso-PGF2α was only associated with Cs (%Δ= 41.6, 95% CI: 2.5, 95.7) and Zn (%Δ = 53.6, 95% CI: 7.4, 119.6), with wide confidence intervals. Urinary Cu and Zn concentrations were also associated with 9.4% (95% CI: 1.1, 18.3) and 8.2% (95% CI: 0.3, 16.7) increases in 8-iso-PGF2α level, respectively. The IQR increases in Cs and Zn were associated with a 9.4% and 13.1% higher PGF2α levels (Cs 95% CI: 0.8, 18.7; Zn 95% CI: 3.4, 23.7). After adjusting for multiple comparisons, relationships of urinary Cu and Cs, with 8-iso-PGF2α, as well as the chemical fraction of the 8-iso-PGF2α, remained statistically significant. Results from GAMM including metals concentrations as splines and the GAMM output graphics showed that the observed associations are linear when significant, after adjusting for covariates.

### 3.4. Windows of Vulnerability Analysis

**Figure 3:**
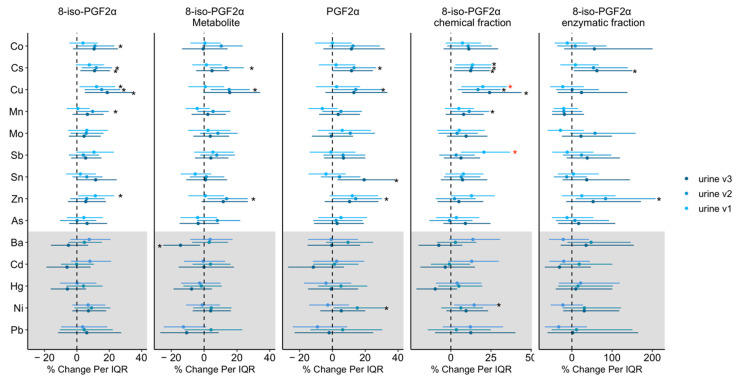
Percent change in prostanoids associated with urinary metal concentrations by study visit. Effect estimates presented as percent changes (%) for interquartile range (IQR) increase in exposure biomarker concentration. Models were adjusted for study visit, maternal age, maternal education, marital status, pre-pregnancy BMI, and exposure to secondhand smoking. Abbreviations: cobalt (Co); cesium (Cs); copper (Cu); manganese (Mn); molybdenum (Mo); antimony (Sb); tin (Sn); zinc (Zn); arsenic (As); barium (Ba); cadmium (Cd); mercury (Hg); nickel (Ni); lead (Pb). White shading indicates essential metals and grey shading indicates non-essential metals. Since Cs is not regarded as essential to the health of plants or animals nor does it present a hazard to them, Cs was considered as essential metal for the analysis. Black * denotes *p* value < 0.05; red * denotes *p* value < 0.05 and *q* value (false discovery rate) < 0.05.

The visit-specific associations between urinary metals and prostanoid markers are shown in Figure 3, and all visit specific estimates, confidence intervals and *p* values are presented in Supplemental Table S2. The effect estimates were generally in the same direction when comparing the three visits. One exception is that Ba at visit 3 was negatively associated with 8-iso-PGF2α metabolite concentration (%Δ/IQR= −14.4, 95% CI: −24.9, −2.4) while the association was null at visit 1 and 2. The differences on the effects estimates of Ba between the visits were significant as *p* value for interaction was 0.04 (visit 3 vs. 1) and 0.03 (visit 3 vs. 2). Although the impact of other urinary metals on oxidative stress did not statistically vary by visits (*p* value for interaction >0.05), a few associations at visit 1 were more robust compared to the other two visits. For example, a significant 20% and 21% increase in the chemical fraction of 8-iso-PGF2α per IQR increase in Cu (%Δ = 19.9, 95% CI: 6.5, 35.0) and Sb (%Δ = 20.6, 95% CI: 6.2, 36.9) at visit 1 were still significant after correction for multiple testing.

### 3.5. Sex-Specific Analysis

**Figure 4:**
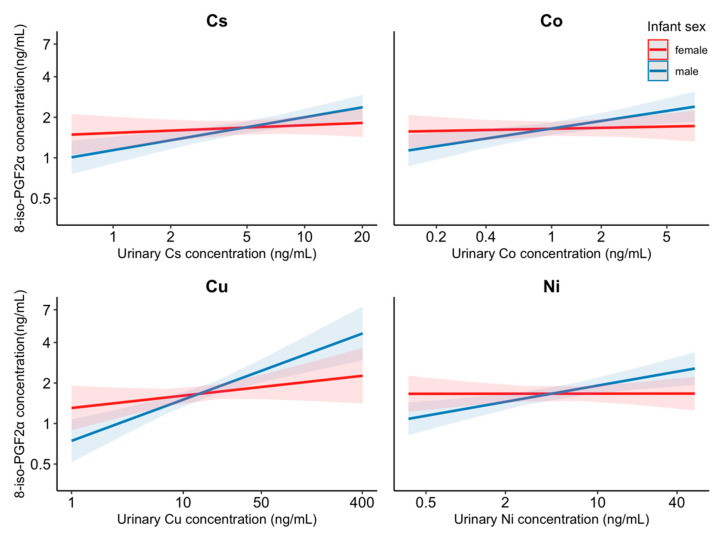
Interaction effect of infant sex on the association between the urinary Cs, Co, Cu, and Ni concentration and 8-iso-PGF2α. Models were adjusted for specific gravity, study visit, maternal age, maternal education, marital status, pre-pregnancy BMI, and exposure to secondhand smoking. Abbreviations: cobalt (Co); cesium (Cs); copper (Cu); nickel (Ni). Lines indicate dose-response curve and shading indicates 95% confidence intervals.

Models with interaction terms between infant sex and metals suggested differences in susceptibility by infant sex for the effects of urinary concentrations of Co, Cs, Cu, and Ni on 8-iso-PGF2α (interaction *p* value = 0.05, 0.05, 0.02, 0.02) and the chemical fraction of the 8-iso-PGF2α (interaction *p* value = 0.03, 0.01, 0.02, 0.01); the associations were only significant among male infants (*p* = 0.003, <0.001, <0.001, 0.001) but not female infants (*p* = 0.71, 0.47, 0.19, 0.99). 14–21% increases in 8-iso-PGF2α associated with one IQR increase in Co (%Δ = 14.3, 95% CI: 4.8, 24.6), Cs (%Δ = 14.4, 95% CI: 6.5, 22.9), Cu (%Δ = 21.2, 95% CI: 11.4, 31.8), and Ni (%Δ = 14.6, 95% CI: 5.5, 24.4) were observed among women who delivered male infants. Figure 4 depicts the modifying effect of infant sex on the association between these metals and 8-iso-PGF2α. Similar differences were observed for the chemical fraction of 8-iso-PGF2α (data not shown).

## 4. Discussion

In this study, we evaluated relationships between urinary concentrations of various metal(loid)s and markers of oxidative stress during pregnancy among Puerto Rican women. After accounting for multiple comparisons, the most robust associations found in this study were between urinary Cs, Cu and increased 8-iso-PGF2α, respectively, with 15% and 11% increases for each IQR increase in Cs and Cu. The additional analysis of Cs and Cu with the fractions thought to reflect 8-iso-PGF2α chemical and enzymatic fractions showed that the magnitude and the significance of the associations with the chemical fraction of the 8-iso-PGF2α is in concordance with 8-iso-PGF2α associations. These findings suggest that the effect of Cs and Cu on 8-iso-PGF2α may be primarily attributable to the chemical lipid peroxidation pathway. Although the chemical and enzymatic fractions have been hypothesized to distinguish the contribution of 8-iso-PGF2α from two pathways, it is worth noting that both inflammation or oxidative stress can lead to the changes in the other as they are interrelated [69,70].

Among women in this study, urinary Cs were higher compared to US women aged 18–40 reported from the National Health and Nutrition Examination Survey (NHANES) [64] but lower than the levels reported among pregnant women in Australia and Spain [71,72]. Cs is an alkali metal that naturally occurs in the environment. Typically, human exposure is low, through inhalation of Cs in the air and/or ingestion of water and food containing Cs [73]. Little is known regarding the health impact of excess Cs exposure. Cs is not regarded as essential to the health of animals or plants, nor is it toxic to them. To our knowledge, no human or animal studies have examined associations between Cs and oxidative stress. However, our findings of urinary Cs associated with higher levels of 8-iso-PGF2α are in line with plant studies that show Cs can induce the formation of ROS and oxidative stress [74,75]. Further studies are needed to assess the mechanisms through which Cs can impact oxidative state in the human body.

Urinary concentrations of Cu among this population were higher compared to NHANES participants [64], but are within the range reported in previous studies of pregnant women in Australia, Spain, and Japan [71,76,77]. Cu plays an essential role in many aspects of human physiology, including acting as cofactor of antioxidant enzymes [78,79]. However, cellular toxicity due to oxidative damage has been linked to excess Cu exposure [80], and some people have increased genetic susceptibility to Cu toxicity (Wilson’s Disease) [81]. Consistent with our findings on Cu, a number of animal and human studies found a relationship between elevated Cu levels and biomarkers of oxidative stress [43,82]. Two different mechanisms have been proposed to explain Cu-induced oxidative damage in the human body: (1) free Cu can catalyze the formation of hydroxyl radicals—powerful reactive oxygen species (ROS) that can damage cellular DNA, membranes and proteins [42,83,84]; and (2) increased levels of Cu may suppress the availability of glutathione, a highly abundant cellular antioxidant [85]. Cu was also associated with higher odds of preterm birth in the Puerto Rican population in our study [38] and in a pregnant women cohort in Boston [48], and it is possible that Cu impacted the early parturition through pathways including oxidative damage.

The window of vulnerability analysis showed positive and robust associations between the chemical fraction of 8-iso-PGF2α and Cu and Sb that are mainly driven by associations in visit 1 (week 18 ± 2 of gestation). Metal(loid)s concentrations may vary across pregnancy due to various factors including their unique physiochemical properties and toxicokinetics, the changes in fetal and maternal nutrient supply [86], and the metabolic changes such as variation in glomerular filtration rate [87,88] and plasma volume expansion [89]. We also showed that the timing of the prenatal visit is important for some of the metal(loid)-oxidative stress associations, including an interaction between Ba concentration and prenatal visit in relation to 8-iso-PGF2α metabolite concentration. The mechanism underlying the negative association between Ba and 8-iso-PGF2α metabolite is unclear as the health effects associated with prenatal Ba are sparsely investigated in the literature. However, the visit-specific results suggest that gestational age may play a critical role in the association between metal(loid)s and oxidative stress.

Our sensitivity analysis of infant sex-specific effects revealed that associations of metals with oxidative stress markers maybe different between women carrying male or female infants. Urinary Co, Cs, Cu, and Ni concentrations measured among pregnant women who delivered male infants were significantly and positively associated with elevated 8-iso-PGF2α levels, whereas the associations were null among women who delivered female infants (Figure 3). Although the differential impact of metals on levels of oxidative stress during pregnancy by fetal sex has not been previously reported, the influences of fetal sex on adverse birth outcomes and the health of pregnant women are becoming better understood. Pregnancy with a male fetus has been associated with higher risk of maternal diabetes, pregnancy complications, maternal sympathetic activation, and placental inflammation [90,91]. There is also evidence for a heightened vulnerability to maternal and/or environmental exposure for male fetuses compared with female fetuses [92,93,94,95]. The sex differential impacts of metals on oxidative stress observed in this study may be attributed, in part, to (1) enzymatic, metabolic, epigenetic differences between male and female fetuses and their interrelation with the maternal environment [90,96] or (2) differences in the hormonal pathways and inflammatory responses involved in mediating effects of infant sex [97,98]. Although the biological mechanisms for these well-documented vulnerabilities remain largely unknown and need further investigation, our findings are suggestive of sex differences in the impact of metals on maternal oxidative stress during pregnancy.

Our study is the first to assess the impact of metals on oxidative stress biomarkers among pregnant women in Puerto Rico. The repeated collection of biological samples enabled us to examine the associations with oxidative stress markers at different times during pregnancy which provided greater statistical power to assess longitudinal associations and potential susceptible windows during pregnancy. While most previous studies evaluated total 8-iso-PGF2α as a biomarker of oxidative stress, we additionally calculated the fraction of chemically-derived 8-iso-PGF2α and enzymatically-derived fraction of 8-iso-PGF2α to distinguish the contribution of the two pathways. The present study does have some limitations. Oxidative stress biomarkers have short half-lives and the levels change over the course of pregnancy [99,100]. However, we did measure markers of both metals and oxidative stress markers at multiple points during pregnancy, increasing the assessment accuracy. While measuring 8-iso PGF2a in the urine provides insight into the systemic state of oxidative stress, it may not represent the redox stress at the placental level where effects from the environmental toxicants may be acting causing preterm birth. Measurement of 8-iso PGF2a at the tissue level may provide more information on the specific oxidative stress occurring here. In this analysis, one of the major assumptions is that metals induce oxidative stress. If oxidative stress causes an increase in urine excretion (i.e., reverse causation), the interpretation of these results would be different. Although this work studied the effect of metals on oxidative stress, other environmental exposures, including phthalates and PAHs, and other mechanisms, such as endocrine disruption, were not explored in this analysis. Future work to investigate the associations between multiple chemical mixture and mechanistic pathways is needed. Additionally, the findings may not be generalizable to other pregnant women populations, considering that the exposure profiles and toxicokinetic of responses to exposure may be quite different compared to pregnant women in Puerto Rico.

## 5. Conclusions

We examined the effect of essential and non-essential metal(loid)s on markers of oxidative stress among pregnant women in Northern Puerto Rico. Results from our study contribute to the growing body of literature suggesting that urinary concentrations of certain metals are associated with elevated levels of oxidative stress during pregnancy, and there is effect modification by fetus sex. This study further highlights the need for future research in this area to examine potential visit-specific and sex-specific effects of environmental exposures on oxidative stress during pregnancy.

## Figures and Tables

**Table 1:** Demographic characteristics of *n* = 215 pregnant women from Puerto Rico.

Variable	Mean (SD)	
maternal age	26.7 (5.5)	
Characteristic	Category	Count (Percent)
Insurance type	private	124 (57.7%)
	public (mi salud)	80 (37.2%)
	missing	11 (5.1%)
maternal education	≤high school/ged	50 (23.3%)
	some college or technical school	73 (34%)
	college degree	64 (29.8%)
	master’s degree or higher	26 (12.1%)
	missing	2 (0.9%)
household income	<$10,000	59 (27.4%)
	≥$10,000 to <$30,000	60 (27.9%)
	≥$30,000 to <$50,000	42 (19.5%)
	≥$50,000	25 (11.6%)
	missing	29 (13.5%)
marital status	single	51 (23.7%)
	married or living together	162 (75.3%)
	missing	2 (0.9%)
parity (# birth)	0	84 (39.1%)
	1	77 (35.8%)
	>1	52 (24.2%)
	missing	2 (0.9%)
infant sex	female	88 (40.9%)
	male	114 (53%)
	missing	13 (6%)
prepregnancy BMI (kg m^−2^)	≤25	105 (48.8%)
	>25 to ≤30	66 (30.7%)
	>30	33 (15.3%)
	missing	11 (5.1%)
employment status	employed	123 (57.2%)
	unemployed	90 (41.9%)
	missing	2 (0.9%)
smoking	never	174 (80.9%)
	ever	36 (16.7%)
	current	3 (1.4%)
	missing	2 (0.9%)
exposure to secondhand smoking	none	186 (86.5%)
	up to 1 h	8 (3.7%)
	more than 1 h	14 (6.5%)
	missing	7 (3.3%)
alcohol consumption	none	92 (42.8%)
	before pregnancy	109 (50.7%)
	within the last few months	12 (5.6%)
	missing	2 (0.9%)

**Table 2:** Urinary concentrations of metal(loid)s (ng/mL) and oxidative stress biomarkers (ng/mL) in 215 pregnant women from Puerto Rico ^1^.

Metal(loid) ^2^	LOD	% >LOD	GM	GSD	25%	50%	75%	95%	Max
Co	0.05	100	1.1	1.6	0.80	1.0	1.4	2.6	8.2
Cs	0.01	100	5.4	1.4	4.3	5.3	6.6	10.0	18.4
Cu	2.5	99.3	15.3	1.5	11.9	14.9	18.7	32.0	149
Mn	0.08	100	1.4	1.6	1.08	1.4	1.7	3.1	31.6
Mo	0.3	100	61.9	1.7	44.7	63.3	84.3	147.6	307
Sb	0.04	90	0.09	1.8	0.07	0.09	0.13	0.23	1.2
Sn	0.1	100	2.0	2.6	1.0	1.7	3.2	11.2	81.4
Zn	2	100	301	1.9	203	327	481	798	2136
As	0.3	100	11.3	2.2	6.6	10.9	17.8	43.1	128
Ba	0.1	99.3	2.4	2.4	1.4	2.4	4.4	10.2	35
Cd	0.06	74.5	0.13	2.3	0.07	0.12	0.20	0.59	7.6
Hg	0.05	98.6	0.56	2.7	0.30	0.58	1.1	2.8	13.6
Ni	0.8	98.9	5.1	1.7	3.8	5.2	7.1	12.3	32
Pb	0.1	72.1	0.24	2.4	0.1	0.26	0.41	1.0	4.6
Oxidative stress Biomarkers ^2^		% >LOD	GM	GSD	25%	50%	75%	95%	Max
8-iso-PGF2α		100	1.8	1.9	1.3	2.0	2.9	4.6	11.7
8-iso-PGF2α metabolite		100	0.91	1.8	0.62	0.93	1.4	2.2	7.1
PGF2α		100	2.8	2.1	1.9	2.9	4.5	8.3	40.8

^1^ Abbreviations: cobalt (Co); cesium (Cs); copper (Cu); manganese (Mn); molybdenum (Mo); antimony (Sb); tin (Sn); zinc (Zn); arsenic (As); barium (Ba); cadmium (Cd); mercury (Hg); nickel (Ni); lead (Pb); limit of detection (LOD); geometric mean (GM); geometric standard deviation (GSD). ^2^ Includes specific gravity-corrected urinary metal and oxidative stress biomarkers concentrations for up to three repeated samples per woman (*n* = 337 samples, *n* = 215 women among which 28 have all three measurements, 66 have two measurements, and 121 have one measurement).

## Data Availability

Data utilized for this analysis can be obtained by reasonable request by contacting both the first author (P.A., pahriya@umich.edu) and the corresponding author (J.D.M., meekerj@umich.edu).

## Supplementary Materials

The following are available online at https://www.mdpi.com/2076-3921/10/1/114/s1, Table S1. Percent change in urinary 8-iso-PGF2α, 8-iso-PGF2α metabolite, PGF2α, 8-iso-PGF2α chemical fraction, and 8-iso-PGF2α enzymatic fraction associated with exposure biomarker concentration, Table S2. Percent change in 8-iso-PGF2α, 8-iso-PGF2α metabolite, PGF2α, 8-iso-PGF2α chemical fraction, and 8-iso-PGF2α enzymatic fraction associated with urinary metal biomarker concentration at each visit during pregnancy.
