# Supplementary material for: Maternal Urinary Metal and Metalloid Concentrations in Association with Oxidative Stress Biomarkers
Source: Antioxidants (Basel). 2021 Jan 15;10(1):114. doi: 10.3390/antiox10010114 (PMC7830802; doi:10.3390/antiox10010114)
Supplement: Supplementary file 1 [file antioxidants-10-00114-s001.pdf]

<u>Supplemental Information</u>

Maternal Urinary Metal and Metalloid levels in Association with Oxidative Stress Biomarkers in Northern Puerto Rico

Pahriya Ashrap, Deborah J. Watkins, Ginger L. Milne, Kelly K. Ferguson, Rita Loch-Caruso, Jennifer Fernandez, Zaira Rosario, Carmen M. Vélez-Vega, Akram Alshawabkeh, José F Cordero, John D. Meeker

Table 1. Percent change in urinary 8-iso-PGF2α, 8-iso-PGF2α metabolite, PGF2α, 8-iso-PGF2α chemical fraction, and 8-iso-PGF2α enzymatic fraction associated with exposure biomarker concentration. Effect estimates presented as percent changes for IQR increase in exposure biomarker concentration. Models were adjusted for specific gravity, study visit, maternal age, maternal education, marital status, pre-pregnancy BMI, and exposure to secondhand smoking.

**Metals**	**8-iso-PGF2α**		**8-iso-PGF2α metabolite**		**PGF2α**		**8-iso-PGF2α chemical fraction**		**8-iso-PGF2α enzymatic fraction**	
	**%Δ (95% CI)**	**p value**	**%Δ (95% CI)**	**p value**	**%Δ (95% CI)**	**p value**	**%Δ (95% CI)**	**p value**	**%Δ (95% CI)**	**p value**
Essential Metals										
Co	**7.8 (1.0, 15.1)**	**0.03***	3.7 (-3.5, 11.4)	0.32	6.8 (-2, 16.4)	0.14	**9.3 (0.9, 18.3)**	**0.03***	7.6 (-23.6, 51.6)	0.67
Cs	**11.1 (4.5, 18.2)**	**0.001***	6.6 (-0.5, 14.2)	0.07	**9.4 (0.8, 18.7)**	**0.03***	**14.5 (6.3, 23.4)**	**0.001***	**41.6 (2.5, 95.7)**	**0.04***
Cu	**14.9 (7.2, 23.2)**	**<0.001***	**9.4 (1.1, 18.3)**	**0.03***	9.5 (-0.2, 20.1)	0.06	**19.7 (10.0, 30.3)**	**<0.001***	-5.3 (-34.6, 37.1)	0.77
Mn	4.0 (-1.4, 9.6)	0.16	-0.7 (-6.3, 5.3)	0.82	-1.4 (-8.0, 5.7)	0.70	**7.1 (0.4, 14.2)**	**0.04***	-20.0 (-39.2, 5.4)	0.12
Mo	6.0 (-1.5, 14.1)	0.12	5.4 (-2.7, 14.2)	0.20	5.8 (-3.9, 16.5)	0.25	6.9 (-2.2, 16.9)	0.14	15.1 (-21.5, 68.7)	0.47
Sb	**7.3 (0.2, 14.8)**	**0.05***	6.5 (-1.1, 14.7)	0.10	4.8 (-4.1, 14.7)	0.30	**10.4 (1.6, 19.8)**	**0.02***	19.2 (-16.5, 70.0)	0.34
Sn	6.3 (-0.5, 13.4)	0.07	-1.9 (-8.8, 5.6)	0.61	4.5 (-4.0, 13.7)	0.32	7.9 (-0.4, 16.8)	0.07	4.0 (-25.9, 46.1)	0.82
Zn	**8.4 (1.2, 16.2)**	**0.02***	**8.2 (0.3, 16.7)**	**0.04***	**13.1 (3.4, 23.7)**	**0.01***	7.5 (-1.2, 16.9)	0.10	**53.6 (7.4, 120)**	**0.02***
Non-essential Metals										
As	3.9 (-3.6, 12.0)	0.32	-0.2 (-8.1, 8.4)	0.97	3.8 (-5.9, 14.6)	0.46	4.1 (-5.0, 14.1)	0.39	2.9 (-30.4, 52.3)	0.88
Ba	2.7 (-3.7, 9.6)	0.42	-1.9 (-8.6, 5.3)	0.60	3.7 (-4.9, 13.0)	0.42	3.0 (-4.8, 11.5)	0.47	19.7 (-14.7, 68)	0.30
Cd	0.5 (-5.5, 6.8)	0.88	1.6 (-5.1, 8.7)	0.66	-1.5 (-9.2, 6.8)	0.71	1.9 (-5.4, 9.8)	0.62	-6.4 (-31.9, 28.7)	0.69
Hg	-0.5 (-7.4, 7)	0.90	-4.0 (-11.3, 3.9)	0.31	0.2 (-8.8, 10.0)	0.97	-0.3 (-8.7, 8.9)	0.95	15.8 (-20.4, 68.4)	0.44
Ni	**8.2 (1.2, 15.6)**	**0.02***	2.3 (-4.9, 10.1)	0.54	5.5 (-3.3, 15.2)	0.23	**11.0 (2.4, 20.3)**	**0.01***	8.8 (-23.2, 54.2)	0.64
Pb	4.6 (-5.0, 15.2)	0.36	-6.9 (-16.1, 3.3)	0.18	-2.5 (-14.1, 10.7)	0.70	9.4 (-2.7, 22.9)	0.14	-12.2 (-46.8, 45.0)	0.61

Abbreviations: cobalt (Co); cesium (Cs); copper (Cu); manganese (Mn); molybdenum (Mo); antimony (Sb); tin (Sn); zinc (Zn); arsenic (As); barium (Ba); cadmium (Cd); mercury (Hg); nickel (Ni); lead (Pb).

* denotes $p < 0.05$; *denotes $p < 0.05$ & q value (false discovery rate) <0.05.

Table 2. Percent change in urinary 8-iso-PGF2α, 8-iso-PGF2α metabolite, PGF2α, 8-iso-PGF2α chemical fraction, and 8-iso-PGF2α enzymatic fraction associated with urinary metal biomarker concentration at each visit during pregnancy. Effect estimates presented as percent changes (%) for IQR increase in exposure biomarker concentration[a]. Models were adjusted for study visit, maternal age, maternal education, marital status, pre-pregnancy BMI, and exposure to secondhand smoking.

**Metals**	**8-iso-PGF2α**						**8-iso-PGF2α metabolite**					
	**Visit 1**		**Visit 2**		**Visit 3**		**Visit 1**		**Visit 2**		**Visit 3**	
	%Δ (95% CI)	*p* value	%Δ (95% CI)	*p* value	%Δ (95% CI)	*p* value	%Δ (95% CI)	*p* value	%Δ (95% CI)	*p* value	%Δ (95% CI)	*p* value
Essential metals												
Co	3.7 (-4.7, 12.9)	0.40	**11.1 (0.3, 23.2)**	**0.05***	10.6 (-2.4, 25.3)	0.12	0.5 (-8.4, 10.2)	0.92	10.4 (-1.3, 23.5)	0.09	-0.5 (-13.5, 14.3)	0.94
Cs	7.7 (-0.8, 16.8)	0.08	**12.0 (2.9, 21.8)**	**0.01***	**10.9 (2.1, 20.5)**	**0.02***	1.4 (-7.2, 10.7)	0.77	**13.3 (3.4, 24.2)**	**0.01***	4.8 (-4.9, 15.4)	0.34
Cu	**12.2 (1.7, 23.7)**	**0.02***	**15.3 (4.8, 26.8)**	**0.004***	**18.8 (4.9, 34.4)**	**0.01***	0.7 (-9.5, 12.1)	0.90	**15.2 (3.9, 27.8)**	**0.01***	15.6 (-0.5, 34.1)	0.06
Mn	0.6 (-6.3, 8.0)	0.87	**9.7 (0.6, 19.7)**	**0.04***	6.5 (-2.7, 16.6)	0.17	-4.2 (-11.3, 3.4)	0.27	5.4 (-4.1, 16.0)	0.28	2.3 (-7.7, 13.2)	0.67
Mo	6.1 (-5.5, 19.2)	0.32	5.8 (-4.0, 16.5)	0.26	4.4 (-5.0, 14.8)	0.37	2.4 (-9.7, 16.0)	0.72	8.4 (-2.5, 20.4)	0.14	3.7 (-6.6, 15.2)	0.49
Sb	10.6 (-0.5, 22.9)	0.06	4.0 (-4.9, 13.7)	0.40	5.4 (-3.5, 15.2)	0.25	5.3 (-6.1, 18.2)	0.38	7.7 (-2.3, 18.7)	0.14	4.1 (-5.6, 14.8)	0.42
Sn	2.1 (-6.6, 11.8)	0.64	6.1 (-2.8, 15.8)	0.19	11.6 (-0.1, 24.6)	0.05	-5.4 (-14.3, 4.4)	0.27	1.3 (-8.7, 12.3)	0.81	0.8 (-10.9, 14.0)	0.90
Zn	**11.4 (0.8, 23.1)**	**0.04***	6.0 (-4.1, 17.2)	0.26	5.5 (-5.7, 17.9)	0.35	0.7 (-9.6, 12.2)	0.90	**13.6 (2.0, 26.6)**	**0.02***	11.6 (-1.6, 26.7)	0.09
Non-essential metals												
As	4.3 (-6.3, 16.0)	0.44	0.2 (-10.4, 12.0)	0.98	6.3 (-4.8, 18.7)	0.28	-3.9 (-14.4, 7.8)	0.50	8.0 (-4.4, 22.0)	0.22	-3.6 (-14.9, 9.2)	0.56
Ba	7.7 (-4, 20.8)	0.21	4.6 (-5.2, 15.4)	0.37	-5.2 (-16.0, 7.0)	0.39	3.6 (-8.4, 17.3)	0.57	3.1 (-7.3, 14.6)	0.58	**-14.4 (-24.9, -2.4)**	**0.02***
Cd	8.0 (-3.7, 21.2)	0.19	-0.2 (-9.8, 10.5)	0.97	-6.1 (-18.8, 8.5)	0.39	-0.5 (-12.2, 12.8)	0.94	3.9 (-7.0, 16.1)	0.50	-0.1 (-15.5, 18.2)	0.99
Hg	0.2 (-10.5, 12.1)	0.98	4.1 (-6.5, 15.9)	0.47	-5.9 (-16.1, 5.7)	0.31	-2.7 (-13.9, 9.9)	0.66	-1.6 (-12.7, 10.8)	0.78	-7.7 (-18.6, 4.8)	0.22
Ni	6.9 (-2.8, 17.6)	0.17	9.0 (-1.5, 20.7)	0.10	7.1 (-2.9, 18.2)	0.17	-1.2 (-11.0, 9.7)	0.82	4.4 (-6.7, 16.7)	0.45	4.0 (-7.0, 16.3)	0.49
Pb	3.6 (-9.7, 18.9)	0.62	4.6 (-10.5, 22.2)	0.57	6.1 (-11.6, 27.4)	0.53	-12.6 (-24.6, 1.4)	0.08	4.2 (-11.9, 23.2)	0.63	-10.7 (-26.7, 8.8)	0.27

	**PGF2α**						**8-iso-PGF2α chemical fraction**					
	**Visit 1**		**Visit 2**		**Visit 3**		**Visit 1**		**Visit 2**		**Visit 3**	
	%Δ (95% CI)	*p* value	%Δ (95% CI)	*p* value	%Δ (95% CI)	*p* value	%Δ (95% CI)	*p* value	%Δ (95% CI)	*p* value	%Δ (95% CI)	*p* value
Essential metals												
Co	0.0 (-10.6, 11.8)	1.00	12.5 (-1.7, 28.9)	0.09	11.6 (-5.5, 31.9)	0.20	7.2 (-3.3, 18.8)	0.19	10.7 (-2.3, 25.5)	0.11	11.2 (-4.5, 29.5)	0.18
Cs	2.3 (-8.3, 14.0)	0.69	**13.2 (1.2, 26.6)**	**0.03***	11.6 (0.0, 24.7)	0.05	**13.5 (2.8, 25.2)**	**0.01***	**12.9 (2.0, 25.0)**	**0.02***	**12.3 (1.6, 24.1)**	**0.02***
Cu	2.5 (-10.0, 16.9)	0.71	**14.2 (0.6, 29.8)**	**0.04***	13.1 (-4.2, 33.4)	0.15	**19.9 (6.5, 35.0)**	**0.003***	**16.9 (4.2, 31.1)**	**0.01***	**24.1 (6.9, 44.0)**	**0.01***
Mn	-6.1 (-14.6, 3.2)	0.19	5.2 (-6.2, 17.9)	0.39	3.6 (-8.1, 16.8)	0.57	4.9 (-3.8, 14.4)	0.28	**11.4 (0.2, 23.8)**	**0.05***	7.9 (-3.3, 20.4)	0.18
Mo	6.0 (-9.0, 23.5)	0.46	10.9 (-2.3, 26.0)	0.11	-0.7 (-12.5, 12.6)	0.91	5.2 (-8.5, 21.1)	0.48	3.9 (-7.6, 16.8)	0.52	9.5 (-2.4, 22.8)	0.12
Sb	-0.9 (-13.8, 14.0)	0.90	6.5 (-5.5, 20.0)	0.31	6.7 (-5.1, 20.1)	0.28	**20.6 (6.2, 36.9)**	**0.005***	3.3 (-7.3, 15.0)	0.56	6.3 (-4.5, 18.2)	0.26
Sn	-3.9 (-14.6, 8.1)	0.51	4.4 (-7.0, 17.1)	0.47	**19.3 (3.1, 38.1)**	**0.02***	7.8 (-3.4, 20.3)	0.18	6.5 (-4.3, 18.4)	0.25	7.3 (-6.2, 22.7)	0.31
Zn	12.1 (-1.6, 27.7)	0.09	**14.2 (0.1, 30.3)**	**0.05***	10.5 (-4.7, 28.1)	0.19	12.9 (0.0, 27.4)	0.05	2.3 (-9.5, 15.5)	0.72	5.0 (-8.3, 20.2)	0.48
Non-essential metals												
As	5.2 (-8.6, 21.1)	0.48	2.2 (-11.7, 18.4)	0.77	2.9 (-11.2, 19.2)	0.71	3.4 (-9.2, 17.7)	0.61	-0.9 (-13.5, 13.5)	0.90	9.0 (-4.6, 24.7)	0.21
Ba	-0.7 (-14.7, 15.6)	0.92	9.5 (-3.8, 24.7)	0.17	-0.4 (-15.2, 16.9)	0.96	13.7 (-1.1, 30.7)	0.07	2.8 (-8.7, 15.9)	0.65	-7.5 (-20.2, 7.1)	0.30
Cd	2.6 (-11.9, 19.4)	0.74	1.2 (-11.6, 15.9)	0.86	-11.6 (-27, 7.0)	0.21	13.0 (-1.7, 29.9)	0.09	-0.9 (-12.4, 12.0)	0.88	-3.5 (-19.0, 15.0)	0.69
Hg	-3.9 (-17.2, 11.5)	0.60	5.3 (-8.6, 21.3)	0.48	-0.7 (-14.8, 15.7)	0.92	3.9 (-9.3, 19.0)	0.59	5.0 (-7.9, 19.6)	0.47	-9.7 (-21.5, 3.8)	0.15
Ni	-2.8 (-14.2, 10.1)	0.66	**15.2 (0.7, 31.7)**	**0.04***	5.4 (-7.4, 20.0)	0.43	**14.5 (2.0, 28.5)**	**0.02***	6.2 (-6.1, 20.1)	0.34	9.4 (-2.8, 23.2)	0.14
Pb	-9.1 (-24.1, 8.9)	0.30	6.2 (-13.4, 30.4)	0.56	-2.0 (-23, 24.8)	0.87	12.1 (-5.1, 32.5)	0.18	3.4 (-14.4, 24.9)	0.73	12.4 (-9.9, 40.2)	0.30

	8-iso-PGF2α enzymatic fraction					
	**Visit 1**		**Visit 2**		**Visit 3**	
	%Δ (95% CI)	*p* value	%Δ (95% CI)	*p* value	%Δ (95% CI)	*p* value
Essential metals						
Co	-11.2 (-43, 38.2)	0.60	8.5 (-36.6, 85.8)	0.77	55.8 (-19.3, 201)	0.19
Cs	8.8 (-29.0, 66.8)	0.70	53.6 (-1.2, 139)	0.06	**62.1 (5.1, 150)**	**0.03***
Cu	-22.8 (-54.1, 29.8)	0.33	0.8 (-39.1, 66.9)	0.98	23.9 (-35.5, 138)	0.52
Mn	-20.5 (-45.2, 15.4)	0.23	-20.0 (-49.2, 25.8)	0.34	-18.7 (-49.2, 30.2)	0.39
Mo	-28.5 (-60.6, 29.8)	0.27	57.4 (-4.2, 157)	0.08	22.6 (-24.6, 99.5)	0.41
Sb	-11.4 (-49.0, 53.8)	0.67	24 (-22.4, 98.2)	0.37	38.1 (-13.1, 120)	0.18
Sn	4.0 (-35.0, 66.4)	0.87	-12.9 (-44.9, 37.6)	0.56	36.9 (-23.3, 144)	0.29
Zn	24.6 (-25.6, 109)	0.40	**83.7 (9.5, 208)**	**0.02***	52.7 (-14.1, 172)	0.15
Non-essential metals						
As	-12.1 (-49.6, 53.4)	0.65	7.6 (-39.8, 92.4)	0.81	16.8 (-34.3, 108)	0.60
Ba	-21.5 (-56.8, 42.6)	0.43	47.8 (-11.3, 146)	0.14	35.3 (-28.0, 154)	0.35
Cd	-20.4 (-56.2, 44.8)	0.46	18.2 (-30.4, 101)	0.54	-30.7 (-67.4, 47.1)	0.34
Hg	21.7 (-32.3, 119)	0.51	15.2 (-34.2, 102)	0.62	9.6 (-40.1, 100)	0.77
Ni	-22.4 (-52.8, 27.5)	0.32	30.9 (-22.9, 122)	0.32	30.7 (-21.6, 118)	0.31
Pb	-32.8 (-67.1, 37.4)	0.28	11.5 (-50.4, 151)	0.79	2.3 (-60.5, 165)	0.96

Abbreviations: cobalt (Co); cesium (Cs); copper (Cu); manganese (Mn); molybdenum (Mo); antimony (Sb); tin (Sn); zinc (Zn); arsenic (As); barium (Ba); cadmium (Cd); mercury (Hg); nickel (Ni); lead (Pb).

* denotes $p < 0.05$

*denotes $p < 0.05$ & q value (false discovery rate) <0.05
